# Semi-Synthesis of New 1,2,3-Triazole Derivatives of 9-Bromonoscapine and their Anticancer Activities

**DOI:** 10.22037/ijpr.2020.113213.14170

**Published:** 2021

**Authors:** Zahra Hasanpour, Peyman Salehi, Morteza Bararjanian, Mohammad-Ali Esmaeili, Mostafa Alilou, Maryam Mohebbi

**Affiliations:** a *Department of Phytochemistry, Medicinal Plants and Drugs Research Institute, Shahid Beheshti University, Tehran, Iran. *; b *Schulich School of Medicine and Dentistry and Robarts Research Institute, Western University, London, Ontario, Canada. *; c *Institute of Pharmacy, Pharmacognosy, Center for Molecular Biosciences, University of Innsbruck, 6020 Innsbruck, Austria.*

**Keywords:** 9-Bromonoscapine, Triazole, Anti-cancer, Click chemistry, Breast cancer

## Abstract

Novel 1,2,3-triazole-tethered 9-bromonoscapine derivatives were synthesized by the propargylation of N-nornoscapine followed by Huisgen’s 1,3-dipolar cycloaddition of the terminal alkynes with different azides. Cytotoxicity of the products was studied by MTT assay against the MCF-7 breast cancer cell line. Most of the compounds revealed a better cytotoxicity than N-nornoscapine and 9-bromonornoscapine as the parent compounds. Among the synthesized compounds, those with a hydroxylated aliphatic side chain (5p, 5q, and 5r) showed the highest activities (IC50s: 47.2, 37.9, and 32.3 μg/mL, respectively). Molecular docking studies showed that these compounds also had the highest docking scores and effective interactions with binding sites equal to -8.074, -7.425 and -7.820 kcal/mol, respectively.

## Introduction

In 1817, P. J. Robiquet isolated noscapine from *Papaver Somniferum,* which showed properties unlike other opium alkaloids extracted from papaveraceae family (1). Previous researches implied that noscapine exhibits some bioactivities such as antineoplastic and antitussive (2, 3). The antitussive property of noscapine is due to its effect on sigma opioid receptors as an agonist with mild painkiller properties. In addition, animal studies showed that the antitussive property of noscapine is comparable to codeine (4). This benzylisoquinoline alkaloid also prevents the progression of leukemia, melanoma, breast cancer, lymphoma, colon cancer, ovarian carcinoma, non-small cell lung cancer, glioblastoma, and prostate cancer. At the same time, it has little or no significant toxicity to other body membranes such as heart, kidney, liver, spleen and small intestine without inhibitory in immune responses of primary humoral in mice (5, 6). 

In studies about compounds showing tubulin-binding properties with oral availability, noscapine was found to be a stoichiometric tubulin-binding molecule (6). This alkaloid changes tubulins conformation upon binding, but they are allowed to be polymerized and form microtubules (6). Ye *et al.* found that noscapine can treat solid lymphoid tumors, melanoma, and human breast tumors grown in nude mice (6). In 2002, Joshi *et al.* showed that noscapine could significantly prevent the assembly of tubulin in high efficacy (7). To approve this fact, *in-vitro* tests showed that microtubules were produced in concentrations higher than 100 μM, while many other anti-cancer compounds, such as paclitaxel and vinblastine as microtubule inhibitors, strongly promote assembling of microtubule or inhibition of them in high concentration (7).

**Figure 1:**
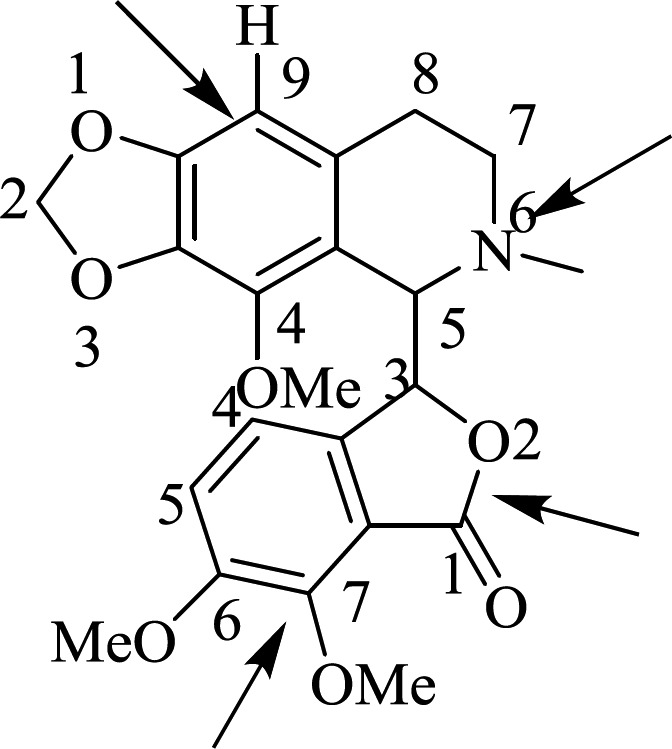
Noscapine

Various researchers have semi-synthesized analogs of noscapine to discover their biological activities (8). Some new derivatives were synthesized in these researches to manipulate different positions such as 1, 7, 9, and 6 (Figure 1) (9). 

In 2002 Egarval *et al.,* and in 2012, Debono *et al*. synthesized some *N*-substituted derivatives of nor-noscapine, such as *N*-carbamate, °*N*-thiocarbamoyl, °*N*-alkyl, °*N*-carbamoyl, and °*N*-acyl derivatives (10, 11). The produced derivatives were able to improve the anti-cancer activity of the parent compound (10). Another modification was a substitution of hydrogen in position 9 by halogens, amine, and azide groups (12).

Biological tests have proved that halogenated analogs (9^′^-halonoscapines; halo: F, Cl, Br, I) had a higher associated affinity for tubulin in comparison with noscapine (13, 14). All noscapine halogenated derivatives, except 9-iodonoscapine, were more active than noscapine in terms of the proliferation of cancer cells (9). Their IC_50_ values in three cell lines, including MCF-7, MDA-MB-231, and CEM were better than noscapine (8, 15). Amazingly, 9-Br-noscapine showed higher cytotoxic activity (IC_50 _= 1.0 ± 0.2 μM) against growth of the MCF-7 cells 40 times more than the parent noscapine and *N*-nornoscapine (IC_50 _= 39.6 ± 2.2 and >100 μM, respectively) (8, 13, 16, 17). According to these results, new derivatives based on 9′-halo noscapine have been interesting targets for semi-synthesis in which 9-bromo derivatives were among the most active compounds. In another study, some Strecker derivatives of *N*-nornoscapine displayed considerable antiparasitic property on Trypanosoma brucei rhodesiense and Plasmodium falciparum (18)*. *Also, these new derivatives were shown to be effective on fibrillation of insulin (19). Triazoles are famous heterocycles that show noticeable properties including anti-viral, anti-microbial, anti-fungal and anti-oxidant (20). In the special case of noscapine, some of the mentioned properties were improved by putting 1,2,3-triazolyl glycoconjugates in the position 7 (2). In doing so, researchers have considered the capability of 1,3-dipolar Huisgen’s cycloaddition reactions to produce two different 1,4 and 1,5 disubstituted 1,2,3-triazoles (21). The meaning of click chemistry proposed by Sharpless *et al.* in 2001, in this paper is a fast, selective, high yield, and green reaction (21, 22). Another property that makes triazoles attractive for many medicinal chemists is their resemblance with amide bonds in electron cloud and atomic distance (20). According to these properties and synergistic effects, new 1,2,3-triazole hybrid derivatives with natural products such as noscapine, curcumin and amino acids have been designed to find new prodrugs with the special properties (23-26). 

Here, we report the synthesis of some new 1,2,3-triazole tethered 9-bromonornoscapine derivatives and investigate their cytotoxic properties.

## Experimental

*General*

Utilized materials, including silica gel and TLC sheets, were purchased from Merck and Sigma-Aldrich companies. Noscapine was used without further purification and donated by the Faran Shimi Pharmaceutical Co. Measurement of the melting point was carried out using a Barnstead-Electrothermal 9200 instrument. IR spectra were recorded on a Bruker Tensor 27 device. NMR spectra were recorded by Bruker Avance III devices 300 and 600 MHz. High resolution MS was obtained by Bruker micro TOF-Q mass spectrometer.

Azides were synthesized according to the recorded methods in the literature (27, 28). Also, nor-noscapine (**2**), was synthesized using the method described in previous reports (29, 30).

*Chemistry*

*Synthetic procedures*

*General procedure for the preparation of 9-Bromo-nor-noscapine *(**3**)

Nor-noscapine (1 g, 2.42 mmol) was dissolved in HBr (48%, 2 mL) and stirred at room temperature. Immediately, a freshly prepared aqua solution of bromine (2%) was added dropwise to the reaction mixture until a yellow precipitate appeared. The reaction was completed at room temperature in 1 h. Ammonia solution (25%) was added to adjust pH at 9-10 and, the mixture was extracted with chloroform (3 × 80 mL). Organic layers were mixed and dried by MgSO_4_. The solvent was removed under reduced pressure, and the crude product was used in the next step without further purification. 

*(S)-3-((R)-9-bromo-4-methoxy-5,6,7,8-tetrahydro-[1,3]dioxolo[4,5-g]isoquinolin-5-yl)-6,7-dimethoxyisobenzofuran-1(3H)-one (3, C*_21_ *H*_20_ *BrNO*_7_ *)*

 Yield: 85%, orange solid, m.p.:188-190 ^o^C, HRMS: [M+H]^+ ^calcd =478.4232, found=479.4522 , IR (KBr, cm^-1^): 3446, 2928, 1758, 1609, 1497, 1449, 730, ^1^H-NMR (600 MHz, CDCl_3_) δ (ppm): 1.92-1.99 (m, 1H), 2.16-2.22 (m, 1H), 2.28-2.35 (m, 1H), 2.47-2.55 (m, 1H), 3.83 (s, 3H), 3.98 (s, 3H), 4.07 (s, 3H), 4.79 (d, *J = *4.0 Hz, 1H), 5.87 (d, 1H, *J = *4.0 Hz, 1H), 6.01 (d, *J = *8.3 Hz, 1H), 6.04 (s, 2H), 6.94 (d, *J = *8.3 Hz, 1H), ^13^C-NMR (150 MHz, CDCl_3_) δ ppm: 28.9, 39.2, 52.9, 56.8, 59.7, 62.3, 80.3, 96.6, 101.2, 117.4, 118.5, 119.2, 119.4, 130.7, 134.4, 139.8, 141.0, 146.6, 148.1, 152.3, 168.3.

*Synthesis of N-propargyl 9-bromo-nor-noscapine *(**4**)

**3** (1.89 mmol, 0.9 g) was dissolved in acetonitrile (8 mL). Then, potassium carbonate (2 equiv, 3.78 mmol, 0.522 g) and propargyl bromide 80% in toluene (1.2 equiv, 2.26 mmol, 0.0028 g) were added. The mixture was refluxed for 8 h. Acetonitrile was removed by rotary evaporator, and the residue was extracted with CHCl_3 _(3×80mL). Finally, the crude material was purified by flash column chromatography on silica gel with CH_2_Cl_2_ as the mobile phase.

*(S)-3-((R)-9-bromo-4-methoxy-6-(prop-2-yn-1-yl)-5,6,7,8-tetrahydro-[1,3]dioxolo[4,5-g]isoquinolin-5-yl)-6,7-dimethoxyisobenzofuran-1(3H)-one (4, C*_24_ *H*_22_ *BrNO*_7_ *)*

 Yield: 80% , yellow powder, m.p.: 175-177 ^o^C, HRMS: [M+H]^+^ calcd= 516.0574, found= 516.0640, IR (KBr, cm^-1^): 3290, 2945, 2840, 1760, 1609, 1497, 1447, 1387, 1267, 1215, 1042, 907, 803, 730, 645, ^1^H-NMR (600 MHz, CDCl_3_) δ (ppm): 1.91-2.02 (m, 1H), 2.42-2.49 (m, 2H), 2.57-2.67 (m, 2H), 2.74-2.83 (m, 1H), 2.93-3.01 (m, 1H), 3.87 (s, 3H), 3.99 (s, 3H), 4.08 (s, 3H), 4.27 (d, *J = *4.7 Hz, 1H), 5.39 (d, *J = *4.7 Hz, 1H), 6.02 (s, 2H), 6.26 (d, *J = *8.2 Hz, 1H), 6.96 (d, *J = *8.2 Hz, 1H), ^13^C-NMR (150 MHz, CDCl_3_) δ (ppm): 27.3, 46.2, 46.6, 56.8, 57.5, 59.7, 62.3, 72.5, 79.9, 81.6, 95.51, 101.2, 117.6, 118.2, 119.3, 120.0, 131.1, 134.6, 139.8, 140.4, 146.6, 147.7, 152.4, 167.8.

*General procedure for preparation of 1,2,3-triazole derivatives (* ***5a*** *-* ***5t)***

**4** (0.080 g, 0.15 mmol) was dissolved in 1 mL solvent that was a mixture of methanol: dichloromethane: water (1: 1: 1). Then 10 mol% of CuSO_4_.5H_2_O (0.015 mmol, 0.0037 g) and 20 mol% of sodium ascorbate (0.03 mmol, 0.006 g) were added to the flask. The reaction mixture was stirred at room temperature for 10 min until complete consumption of **4,** which was confirmed by TLC (toluene: ethyl acetate, 2:1). Ammonia solution (25%) was added, and the crude product was extracted with CH_2_Cl_2_ (3×10 mL). Finally, 1,2,3-triazole derivatives were purified by preparative thin layer chromatography (toluene: ethyl acetate, 3:1).

*(S)-3-((R)-9-bromo-6-((1-(3,4-dimethoxyphenyl)-1H-1,2,3-triazol-4-yl)methyl)-4-methoxy-5,6,7,8-tetrahydro-[1,3]dioxolo[4,5-g]isoquinolin-5-yl)-6,7-dimethoxyisobenzofuran-1(3H)-one (5a, C*_32_ *H*_31_ *BrN*_4_ *O*_7_ *)*

 Yield: 85%, yellow powder, m.p.: 172-174 ^o^C, HRMS: [M+H]^+^ calcd= 696.1269, found= 697.1861, IR (KBr, cm^-1^): 3450, 2942, 2842, 1755, 1607, 1510, 1450, 730, ^1^H-NMR (600 MHz, CDCl_3_) δ (ppm): 1.92-2.04 (m, 1H), 2.12-2.20 (m, 1H), 2.44-2.53 (m, 1H), 2.55-2.62 (m, 1H), 3.81 (d, *J = *14.1 Hz, 1H), 3.84 (s, 3H), 3.92 (s, 3H), 4.00 (s, 3H), 4.02 (s, 3H), 4.03 (d, *J = *14.1 Hz, 1H), 4.05 (s, 3H), 4.49 (d, *J = *4.2 Hz, 1H), 5.72 (d, *J = *4.2 Hz, 1H), 6.03 (s, 2H), 6.13 (d, *J = *8.2 Hz, 1H), 6.95 ( d, *J = *8.6 Hz, 1H), 6.96 (d, *J = *8.2 Hz, 1H), 7.38 (dd, *J = *8.6, 2.4 Hz, 1H), 7.49 (d, *J = *2.4 Hz, 1H), 8.24 (s, 1H), ^13^C-NMR (150 MHz, CDCl_3_) δ (ppm): 24.5, 45.8, 52.2, 56.3, 56.7, 56.7, 58.5, 59., 62.3, 80.2, 96.0, 101.2, 104.5, 111.3, 112.1, 117.7, 118.3, 119.4, 122.1, 130.2, 131.0, 134.3, 140.0, 140.5, 146.7, 146.8, 148.0, 149.0, 149.7, 152.5, 168.7.

*(S)-3-((R)-9-bromo-4-methoxy-6-((1-(4-methoxyphenyl)-1H-1,2,3-triazol-4-yl)methyl)-5,6,7,8-tetrahydro-[1,3]dioxolo[4,5-g]isoquinolin-5-yl)-6,7-dimethoxyisobenzofuran-1(3H)-one (5b, C*_31_ *H*_29_ *BrN*_4_ *O*_8_ *)*

 Yield: 90%, yellow powder, m.p.: 152-154^o^C, HRMS: [M+H]^+^ calcd= 665.1163, found= 667.1715, IR (KBr, cm^-1^): 3449, 2925, 2851, 1754, 1613, 1509, 1449, 730, ^1^H-NMR (300 MHz, CDCl_3_) δ (ppm): 1.96-2.01 (m, 1H), 2.21-2.29 (m, 1H), 2.52-2.59 (m, 2H), 3.83 (d, *J = *13.02 Hz, 1H), 3.87 (s, 3H), 3.88 (s, 3H), 4.05 (s, 3H), 4.06 (d, *J = *13.0 Hz, 1H), 4.11 (s, 3H), 4.55 (d, *J = *4.0 Hz, 1H), 5.73 (d, *J = *4.0 Hz, 1H), 6.06 (s, 2H), 6.17 (d, *J = *8.2 Hz, 1H), 6.99 (d, *J = *8.2 Hz, 1H), 7.05 (d , *J = *9.0 Hz, 2H), 7.80 (d, *J = *9.0 Hz, 2H), 8.22 (s, 1H). 

*(S)-3-((R)-9-bromo-4-methoxy-6-((1-(p-tolyl)-1H-1,2,3-triazol-4-yl)methyl)-5,6,7,8-tetrahydro-[1,3]dioxolo[4,5-g]isoquinolin-5-yl)-6,7-dimethoxyisobenzofuran-1(3H)-one (5c, C*_31_*H*_29_*BrN*_4_*O*_7_*)* Yield: 85%, yellow powder, m.p.: 113-116 ^o^C, HRMS: [M+H]^+^ calcd= 649.1214, found= 649.1348, IR (KBr, cm^-1^): 3451, 2930, 2842, 2358, 1756, 1611, 1502, 1443, 813, 730. ^1^H-NMR (600 MHz, CDCl_3_) δ (ppm): 1.89-1.95 (m, 1H), 2.20-2.27 (m, 1H), 2.40 (s, 3H), 2.42-2.50 (m, 1H), 2.52-2.61 (m, 1H), 3.83 (s, 3H), 3.84 (d, *J = *14.0 Hz, 1H), 4,00 (s, 3H), 4.06 (d, *J = *14.0 Hz, 1H), 4.05 (s, 3H), 4.52 (d, *J = *4.3 Hz, 1H), 5.67 (d, *J = *4.3 Hz, 1H), 6.02 (s, 2H), 6.14 (d, *J = *8.2 Hz, 1H), 6.95 (d, *J = *8.2 Hz, 1H), 7.29 (d , *J = *8.2 Hz, 2H), 7.72 (d, *J = *8.2 Hz, 2H), 8.21 (s, 1H), ^13^C-NMR (150 MHz, CDCl_3_) δ (ppm): 21.1, 24.8, 45.7, 52.0, 56.7, 58.6, 59.6, 62.3, 80.4, 95.9, 101.1, 117.7, 118.2, 118.4, 119.8, 120.1, 121.6, 130.1, 130.3, 134.3, 135.0, 138.4, 140.0, 140.5, 146.5, 146.8, 147.9, 152.5, 168.6.

*(S)-3-((R)-9-bromo-4-methoxy-6-((1-phenyl-1H-1,2,3-triazol-4-yl)methyl)-5,6,7,8-tetrahydro-[1,3]dioxolo[4,5-g]isoquinolin-5-yl)-6,7-dimethoxyisobenzofuran-1(3H)-one (5d, C*_30_ *H*_27_ *BrN*_4_ *O*_7_ *)*

 Yield: 85%, yellow powder, m.p.: 97-99 ^o^C, HRMS: [M+H]^+^ calcd= 635.1058, found= 636.8440, IR (KBr, cm^-1^): 3448, 2927, 2357, 1755, 1606, 1500, 1444, 754, ^1^H-NMR (600 MHz, CDCl_3_) δ (ppm): 1.91-1.99 (m, 1H), 2.21-2.27 (m, 1H), 2.48-2.51 (m, 1H), 2.52-2.57 (m, 1H), 3.83 (s, 3H), 3.84 (s, *J = *13.0 Hz, 1H), 4.00 (s, 3H), 4.05 (s, 3H), 4.06 (d, *J = *13.0 Hz, 1H), 4.51 (d, *J = *4.1 Hz, 1H), 5.68 (d, *J = *4.1 Hz, 1H), 6.02 (s, 2H), 6.14 (d, *J = *8.3 Hz, 1H), 6.95 (d, *J = *8.3 Hz, 1H), 7.39 (t, *J = *7.4 Hz, 1H), 7.47-7.54 (m , 2H), 7.86 (d, *J = *7.6 Hz, 2H), 8.27 (s, 1H), ^13^C-NMR (150 MHz, CDCl_3_) δ (ppm): 24.7, 45.7, 52.1, 56.7, 58.5, 59.6, 62.3, 80.4, 95.9, 101.1, 117.7, 118.3, 118.3, 119.8, 120.2, 121.7, 128.4, 129.7, 130.3, 134.3, 137.3, 140.0, 140.5, 146.7, 146.8, 148.0, 152.5, 168.7.

*(S)-3-((R)-9-bromo-6-((1-(4-ethylphenyl)-1H-1,2,3-triazol-4-yl)methyl)-4-methoxy-5,6,7,8-tetrahydro-[1,3]dioxolo[4,5-g]isoquinolin-5-yl)-6,7-dimethoxyisobenzofuran-1(3H)-one (5e, C*_32_ *H*_31_ *BrN*_4_ *O*_7_ *)*

Yield: 85%, yelow powder, m.p.: 98-100 ^o^C, decompose, HRMS: [M+H]^+^ calcd= 663.1371, found= 663.1462, IR (KBr, cm^-1^): 3442, 2924, 2358, 1754, 1621, 1499, 1448, 801, 719. ^1^H-NMR (300 MHz, CDCl_3_) δ (ppm): 0.87 (q, *J = *7.6 Hz, 2H), 1.29 (t, *J = *7.6 Hz, 3H), 1.95-2.02 (m, 1H), 2.22-2.28 (m, 1H), 2.50-2.58 (m, 1H), 2.71-2.77 (m, 1H), 3.90 (s, 3H), 3.99 (d, *J = *14.0 Hz, 1H), 4.04 (s, 3H), 4.08 (d, *J = *14.0 Hz, 1H), 4.10 (s, 3H), 4.56 (d, *J = *3.0 Hz, 1H), 5.72 (d, *J = *3.0 Hz, 1H), 6.06 (s, 2H), 6.26 (d, *J = *9.0 Hz, 1H), 7.00 (d, *J = *9.0 Hz, 1H), 7.38 (d, *J = *9.0 Hz, 2H), 7.79 (d, *J = *9.0 Hz, 2H), 8.26 (s, 1H).

*(S)-3-((R)-9-bromo-4-methoxy-6-((1-(pyridin-3-yl)-1H-1,2,3-triazol-4-yl)methyl)-5,6,7,8-tetrahydro-[1,3]dioxolo[4,5-g]isoquinolin-5-yl)-6,7-dimethoxyisobenzofuran-1(3H)-one (5f, C*_29_ *H*_26_ *BrN*_5_ *O*_7_ *)*

 Yield: 90%, yellow powder, m.p.: 107-109 ^o^C, HRMS: [M+H]^+^ calcd= 636.1010, found= 637.9719, IR (KBr, cm^-1^): 3452, 2925, 2358, 1749, 1618, 1498, 1448, 1268, 1035, 803, 603, ^1^H-NMR (600 MHz, CDCl_3_) δ (ppm): 2.94-1.98 (m, 1H), 2.14-2.20 (m, 1H), 2.44-2.50 (m, 1H), 2.52-2.58 (m, 1H), 3.82 (d, *J = *13.0 Hz, 1H), 3.83 (s, 3H), 4.03 (s, 3H), 4.07 (s, 3H), 4.08 (d, *J = *13.0 Hz, 1H), 4.49 (d, *J = *4.0 Hz, 1H), 5.70 (d, *J = *4.0 Hz, 1H), 6.03 (s, 2H), 6.10 (d, *J = *8.2 Hz, 1H), 6.95 (d, *J = *8.2 Hz, 1H), 7.44-7.52 (m, 1H), 8.20-8.27 (m, 1H), 8.36 (s, 1H), 8.67 (dd, *J = *4.8, 1.3 Hz, 1H), 9.20 (d, *J = *2.5 Hz, 1H), ^13^C-NMR (150 MHz, CDCl_3_) δ (ppm): 24.6, 45.9, 52.1, 56.7, 58.6, 59.7, 62.3, 80.3, 96.0, 101.2, 117.7, 118.1, 118.3, 119.9, 121.9, 124.2, 127.6, 128.8, 130.3, 130.9, 133.8, 134.3, 140.0, 146.9, 147.4, 148.0, 149.5, 152.5, 168.8.

*(S)-3-((R)-9-bromo-4-methoxy-6-((1-(4-nitrophenyl)-1H-1,2,3-triazol-4-yl)methyl)-5,6,7,8-tetrahydro-[1,3]dioxolo[4,5-g]isoquinolin-5-yl)-6,7-dimethoxyisobenzofuran-1(3H)-one (5g, C*_30_ *H*_26_ *BrN*_5_ *O*_9_ *)*

 Yield: 60%, orange powder, m.p.: 108-110 ^o^C, HRMS: [M+H]^+^ calcd= 680.0908, found= 680.0992, IR (KBr, cm^-1^): 3450, 2940, 1755, 1608, 1498, 1448, 730, ^1^H-NMR (300 MHz, CDCl_3_) δ (ppm): 2.02-2.10 (m, 2H), 2.51-2.58 (m, 2H), 3.82 (d, *J = *12.0 Hz, 1H), 3.85 (s, 3H), 4.04 (d, *J = *12.0 Hz, 1H), 4.08 (s, 3H), 4.12 (s, 3H), 4.47 (d, *J = *3.0 Hz, 1H), 5.79 (d, *J = *3.0 Hz, 1H), 6.08 (s, 2H), 6.15 (d, *J = *8.5 Hz, 1H), 7.08 (d, *J = *8.5 Hz, 1H), 8.23 (d, *J = *9.0 Hz, 2H), 8.46 (d, *J = *9.0 Hz, 2H), 8.56 (s, 1H).

*(S)-3-((R)-9-bromo-6-((1-(3,4-dichlorophenyl)-1H-1,2,3-triazol-4-yl)methyl)-4-methoxy-5,6,7,8-tetrahydro-[1,3]dioxolo[4,5-g]isoquinolin-5-yl)-6,7-dimethoxyisobenzofuran-1(3H)-one (5h, C*_30_ *H*_25_ *BrCl*_2_ *N*_4_ *O*_7_ *)*

 Yield: 90%, yellow powder, m.p.: 163-165 ^o^C, HRMS: [M+H]^+^ calcd= 704.0356, found= 706.1173, IR (KBr, cm^-1^): 3448, 2942, 1754, 1604, 1494, 1447, 730, ^1^H-NMR (300 MHz, CDCl_3_) δ (ppm): 1.90-2.03 (m, 1H), 2.12-2.20 (m, 1H), 2.48-2.53 (m, 2H), 3.85 (d, *J = *12.0 Hz, 1H), 3.88 (s, 3H), 4.02 (d, *J = *12.0 Hz, 1H), 4.07 (s, 3H), 4.11 (s, 3H), 4.51 (d, *J = *3.0 Hz, 1H), 5.75 (d, *J = *3.0 Hz, 1H), 6.07 (s, 2H), 6.16 (d, *J = *9.0 Hz, 1H), 7.00 (d, *J = *9.0 Hz, 1H), 7.24 (d, *J = *9.0 Hz, 2H), 7.83 (d, *J = *9.0 Hz, 2H), 8.13 (s, 1H).

*(S)-3-((R)-9-bromo-6-((1-(4-fluorophenyl)-1H-1,2,3-triazol-4-yl)methyl)-4-methoxy-5,6,7,8-tetrahydro-[1,3]dioxolo[4,5-g]isoquinolin-5-yl)-6,7-dimethoxyisobenzofuran-1(3H)-one (5i, C*_30_ *H*_26_ *BrFN*_4_ *O*_7_ *)*

 Yield: 85%, yellow powder , m.p.: 182-184^o^C, HRMS: [M+H]^+^ calcd= 653.0963, found= 653.1042, IR (KBr, cm^-1^): 3437, 2925, 2858, 2353, 1744, 1630, 1512, 1444, 827, 730, ^1^H-NMR (600 MHz, CDCl_3_) δ (ppm): 1.91-1.98 (m, 1H), 2.12-2.20 (m, 1H), 2.44-2.50 (m, 1H), 2.52-2.58 (m, 1H), 3.80 (d, *J = *12.0 Hz, 1H), 3.83 (s, 3H), 4.02 (s, 3H), 4.05 (d, *J = *12.0 Hz, 1H), 4.06 (s, 3H), 4.48 (d, *J = *4.0 Hz, 1H), 5.70 (d, *J = *4.0 Hz, 1H), 6.03 (s, 2H), 6.11 (d, *J = *8.2 Hz, 1H), 6.95 (d, *J = *8.2 Hz, 1H), 7.64 (t , *J = *8.3 Hz, 2H), 7.87 (dd, *J = *8.3, 2.2 Hz, 2H), 8.26 (s, 1H), ^13^C-NMR (150 MHz, CDCl_3_) δ (ppm): 24.5, 45.8, 52.1, 56.7, 58.5, 59.6, 62.3, 80.41, 96.0, 101.2, 117.7, 118.2, 118.3, 119.8, 122.0, 122.1, 122.2, 128.8, 130.9, 133.6, 134.3, 140.0, 140.4, 146.8, 147.0, 148.0, 152.5, 168.8.

*(S)-3-((R)-9-bromo-6-((1-(3-fluorophenyl)-1H-1,2,3-triazol-4-yl)methyl)-4-methoxy-5,6,7,8-tetrahydro-[1,3]dioxolo[4,5-g]isoquinolin-5-yl)-6,7-dimethoxyisobenzofuran-1(3H)-one (5j, C*_30_ *H*_26_ *BrFN*_4_ *O*_7_ *)*

 Yield: 90%, yellow powder, m.p.: 168-170^o^C, HRMS: [M+H]^+^ calcd= 653.0963, found= 653.1045, IR (KBr, cm^-1^): 3446, 2924, 2857, 1763, 1620, 1501, 1450, 892, 798, ^1^H-NMR (300 MHz, CDCl_3_) δ (ppm): 1.95-2.01 (m, 1H), 2.18-2.24 (m, 1H), 2.48-2.54 (m, 2H), 3.83 (d, *J = *12.0 Hz, 1H), 3.88 (s, 3H), 4.06 (s, 3H), 4.10 (s, 3H), 4.11 (d, *J = *12.0 Hz, 1H), 4.53 (d, *J = *3.2 Hz, 1H), 5.75 (d, *J = *3.2 Hz, 1H), 6.07 (s, 2H), 6.16 (d, *J = *9.0 Hz, 1H), 7.00 (d, *J = *9.0 Hz, 1H), 7.14 (dd, *J = *8.0, 2.0 Hz, 1H), 7.52 ( dd, *J = *8.0, 6.0 Hz, 1H), 7.73 (d, *J = *7.0 Hz, 1H), 7.74 (d, *J = *6.0 Hz, 1H), 8.36 (s, 1H). 

*(S)-3-((R)-9-bromo-6-((1-(4-bromophenyl)-1H-1,2,3-triazol-4-yl)methyl)-4-methoxy-5,6,7,8-tetrahydro-[1,3]dioxolo[4,5-g]isoquinolin-5-yl)-6,7-dimethoxyisobenzofuran-1(3H)-one (5k, C*_30_ *H*_26_ *Br*_2_ *N*_4_ *O*_7_ *)*

Yield: 90%, yellow powder, m.p.: 176-178 ^o^C, HRMS: [M+H]^+^ calcd= 712.0163, found= 714.3456, IR (KBr, cm^-1^): 3450, 2940, 1755, 1608, 1498, 1448, 731. ^1^H-NMR (600 MHz, CDCl_3_) δ (ppm): 1.91-1.98 (m, 1H), 2.13-2.19 (m, 1H), 2.44-2.50 (m, 1H), 2.51-2.60 (m, 1H), 3.80 (s, *J = *14.0 Hz, 1H), 3.84 (s, 3H), 4,02 (s, 3H), 4.05 (d, *J = *14.0 Hz, 1H), 4.06 (s, 3H), 4.48 (d, *J = *4.1 Hz, 1H), 5.70 (d, *J = *4.1 Hz, 1H), 6.03 (s, 2H), 6.10 (d, *J = *8.2 Hz, 1H), 6.95 (d, *J = *8.2 Hz, 1H), 7.64 (d , *J = *8.8 Hz, 2H), 7.79 (d, *J = *8.8 Hz, 2H), 8.30 (s, 1H), ^13^C-NMR (150 MHz, CDCl_3_) δ (ppm): 24.5, 45.8, 52.1, 56.7, 58.5, 59.7, 62.3, 80.2, 96.0, 101.2, 117.7, 118.1, 118.3, 119.9, 121.7, 121.8, 121.9, 130.3, 132.8, 134.3, 136.3, 139.9, 140.3, 146.9, 147.2, 148.0, 152.5, 168.8.

*(S)-3-((R)-6-((1-benzyl-1H-1,2,3-triazol-4-yl)methyl)-9-bromo-4-methoxy-5,6,7,8-tetrahydro-[1,3]dioxolo[4,5-g]isoquinolin-5-yl)-6,7-dimethoxyisobenzofuran-1(3H)-one (5l, C*_31_ *H*_29_ *BrN*_4_ *O*_7_ *)*

Yield: 90%, yellow powder, m.p.: 93-95 ^o^C, HRMS: [M+H]^+^ calcd= 649.1214, found= 649.1311, IR (KBr, cm^-1^): 3448, 2939, 1758, 1609, 1497, 1448, 726, ^1^H-NMR (600 MHz, CDCl_3_) δ (ppm): 1.87-1.91 (m, 1H), 2.33-2.37 (m, 1H), 2.42-2.47 (m, 1H), 2.50-2.56 (m, 1H), 3.80 (s, *J = *13.0 Hz, 1H), 3.83 (s, 3H), 3.94 (s, 3H), 3.97 (d, *J = *13.0 Hz, 1H), 3.98 (s, 3H), 4.48 (d, *J = *4.4 Hz, 1H), 5.47 (d, *J = *14.8 Hz, 1H), 5.51 (d, *J = *14.8 Hz, 1H), 5.58 (d, *J = *4.4 Hz, 1H), 6.00 (s, 2H), 6.15 (d, *J = *8.2 Hz, 1H), 6.93 (d, *J = *8.2 Hz, 1H), 7.15-7.34 (m , 2H), 7.25-7.34 (m, 3H), 7.63 (s, 1H), ^13^C-NMR (150 MHz, CDCl_3_) δ (ppm): 25.0, 45.35, 51.8, 54.1, 56.7, 58.7, 59.5, 62.3, 80.6, 95.8, 101.1, 117.7, 118.2, 119.7, 122.0, 123.4, 128.0, 128.5, 129.0, 130.39, 134.3, 134.9, 139.9, 140.7, 146.1, 146.7, 147.8, 152.4, 168.3.

*(S)-3-((R)-9-bromo-4-methoxy-6-((1-(4-methylbenzyl)-1H-1,2,3-triazol-4-yl)methyl)-5,6,7,8-tetrahydro-[1,3]dioxolo[4,5-g]isoquinolin-5-yl)-6,7-dimethoxyisobenzofuran-1(3H)-one (5m, C*_32_ *H*_31_ *BrN*_4_ *O*_7_ *)*

 Yield: 80%, yellow powder, m.p.: 78-80^o^C, decompose, HRMS: [M+H]^+^ calcd = 663.1371, found= 663.1500, IR (KBr, cm^-1^): 3446, 3156, 2855, 1754, 1620, 1501, 1450, 1379, 730, ^1^H-NMR (300 MHz, CDCl_3_) δ (ppm): 1.88-1.93 (m, 1H), 2.36 (s, 3H), 2.41-2.48 (m, 1H), 2.57-2.63 (m, 2H), 3.82 (d, *J = *13.0 Hz, 1H), 3.87 (s, 3H), 3.97 (d, *J = *13.0 Hz, 1H), 3.99 (s, 3H), 4.02 (s, 3H), 4.53 (d, *J = *4.0 Hz, 1H), 5.48 (d, *J = *15.0 Hz, 1H), 5.49 (d , *J = *15.0 Hz, 2H), 5.62 (d, *J = *4.0 Hz, 1H), 6.05 (s, 2H), 6.20 (d, *J = *8.2 Hz, 1H), 6.98 ( d, *J = *8.2 Hz, 1H), 7.01-7.42 (m, 4H), 7.65 (s, 1H). 

*(S)-3-((R)-9-bromo-6-((1-(4-fluorobenzyl)-1H-1,2,3-triazol-4-yl)methyl)-4-methoxy-5,6,7,8-tetrahydro-[1,3]dioxolo[4,5-g]isoquinolin-5-yl)-6,7-dimethoxyisobenzofuran-1(3H)-one (5n, C*_31_ *H*_28_ *BrFN*_4_ *O*_7_ *)*

 Yield: 90%, yellow powder, m.p.: 86-88^o^C, HRMS: [M+H]^+^ calcd= 667.1120, found= 667.1217, IR (KBr, cm^-1^): 3447, 2929, 2357, 1757, 1611, 1503, 1445, 795, ^1^H-NMR (600 MHz, CDCl_3_) δ (ppm): 1.83-1.90 (m, 1H), 2.24-2.30 (m, 1H), 2.39-2.45 (m, 1H), 2.50-2.56 (m, 1H), 3.77 (s, *J = *13.0 Hz, 1H), 3.82 (s, 3H), 3.96 (d, *J = *13.0 Hz, 1H), 3.97 (s, 3H), 3.99 (s, 3H), 4.47 (d, *J = *4.3 Hz, 1H), 5.45 (d, *J = *14.0 Hz, 1H), 5.48 (d, *J = *14.0 Hz, 1H), 5.60 (d, *J = *4.3 Hz, 1H), 6.01 (s, 2H), 6.10 (d, *J = *8.2 Hz, 1H), 6.93 (d, *J = *8.2 Hz, 1H), 7.01-7.11 (m, 2H), 7.28 (dd, *J = *8.6, 5.2 Hz, 2H), 7.66 (s, 1H), ^13^C-NMR (150 MHz, CDCl_3_) δ (ppm): 24.9, 45.5, 52.0, 53.3, 56.7, 58.7, 59.6, 62.2, 80.5, 95.9, 101.1, 115.9, 116.0, 117.7, 118.2, 118.4, 119.8, 123.4, 129.9, 130.0, 130.8, 134.3, 139.9, 140.5, 146.4, 146.7, 147.8, 152.4, 168.4.

*(S)-3-((R)-9-bromo-6-((1-(4-bromobenzyl)-1H-1,2,3-triazol-4-yl)methyl)-4-methoxy-5,6,7,8-tetrahydro-[1,3]dioxolo[4,5-g]isoquinolin-5-yl)-6,7-dimethoxyisobenzofuran-1(3H)-one (5o, C*_31_ *H*_28_ *Br*_2_ *N*_4_ *O*_7_ *)*

 Yield: 90%, yellow powder, m.p. : 71-73^o^C, decompose, HRMS: [M+H]^+^ calcd= 727.0319, found= 729.2023, IR (KBr, cm^-1^): 3436, 2926, 2854, 1756, 1614, 1494, 728, ^1^H-NMR (300 MHz, CDCl_3_) δ (ppm): 1.86-1.93 (m, 1H), 2.27-2.34 (m, 1H), 2.47-2.55 (m, 2H), 3.78 (d, *J = *13.0 Hz, 1H), 3.87 (s, 3H), 3.98 (s, 3H), 4.03 (s, 3H), 4.04 (d, *J = *13.0 Hz, 1H), 4.51 (d, *J = *3.1 Hz, 1H), 5.46 (d, *J = *15.0 Hz, 1H), 5.52 (d, *J = *15.0 Hz, 1H), 5.65 (d, *J = *3.1 Hz, 1H), 6.06 (s, 2H), 6.14 (d, *J = *9.0 Hz, 1H), 7.00 (d, *J = *9.0 Hz, 1H), 7.20 ( d, *J = *9.0 Hz, 2H), 7.51 (d, *J = *9.0 Hz, 2H), 7.71 (s, 1H).

*(3S)-3-((5R)-9-bromo-6-((1-(2-hydroxy-2-phenylethyl)-1H-1,2,3-triazol-4-yl)methyl)-4-methoxy-5,6,7,8-tetrahydro-[1,3]dioxolo[4,5-g]isoquinolin-5-yl)-6,7-dimethoxyisobenzofuran-1(3H)-one (5p, C*_32_ *H*_31_ *BrN*_4_ *O*_8_ *)*

 Yield: 80%, yellow powder, mixture of two isomers (50: 50), m.p.: 75-78^o^C decomposed, HRMS: [M+H]^+^ calcd= 679.1320, found= 679.1406, IR (KBr, cm^-1^) 3441, 2925, 2857, 1752, 1613, 1499, 1449, 824, 707, ^1^H-NMR (300 MHz, CDCl_3_, mixture of two isomers, (53:47)) δ (ppm): 1.85-1.96 (m, 2H, mixture of two isomers), 2.46-2.54 (m, 2H, mixture of two isomers), 3.72-3.78 (m, 2H, mixture of two isomers), 3.86 (s, 3H, minor), 3.88 (s, 3H, major), 3.96 (s, 6H, mixture of two isomers), 3.96-4.05 (m, 2H, mixture of two isomers), 4.07 (s, 6H, mixture of two isomers), 4.21-4.28 (m, 1H, minor), 4.44 (d, *J = *4.0 Hz, 1H, major), 4.57-4.63 (m, 2H, mixture of two isomers), 5.31 (bp, 2H, mixture of two isomers), 5.65 (d, *J = *4.0 Hz, 1H, minor), 5.67-5.76 (m, 4H, mixture of two isomers), 5.82 (dd, *J = *4.0 Hz, 2H, major), 6.05 (s, 2H, major), 6.06 (s, 2H, minor), 6.16 (d, *J = *8.5 Hz, 1H, minor), 6.18 (d, *J = *8.5 Hz, 1H, major), 6.97 (d, *J = *8.5 Hz, 1H, minor), 7.90 (s, 1H, major), 6.99 (d, *J = *8.5 Hz, 1H, major), 7.21-7.27 (m, 3H, minor), 7.30-7.42 (m, 6H, major), 7.81 (s, 2H, mixture of two isomers). 

*(S)-3-((R)-9-bromo-6-((1-(2-hydroxy-2-methylpropyl)-1H-1,2,3-triazol-4-yl)methyl)-4-methoxy-5,6,7,8-tetrahydro-[1,3]dioxolo[4,5-g]isoquinolin-5-yl)-6,7-dimethoxyisobenzofuran-1(3H)-one (5q, C*_28_ *H*_31_ *BrN*_4_ *O*_8_ *)*

Yield: 60%, yellow powder, m.p.: 66-68 ^o^C, decomposed, HRMS: [M+H]^+^ calcd= 631.1320, found= 631.1401, IR (KBr, cm^-1^): 3446, 2928, 1754, 1615, 1498, 1449, 728,^ 1^H-NMR (300 MHz, CDCl_3_) δ (ppm): 1.24 (s, 3H), 1.29 (s, 3H), 1.98-2.05 (m, 1H), 2.15-2.21 (m, 1H), 2.50-2.59 (m, 2H), 3.52-3.60 (m, 1H), 3.76 (d, *J = *15.0 Hz, 1H), 3.87 (s, 3H), 3.96 (d, *J = *15.0 Hz, 1H), 4.05 (s, 3H), 4.07 (s, 3H), 4.30 (d, *J = *15.0 Hz, 1H), 4.42 (d, *J = *15.0 Hz, 1H), 4.52 (d, *J = *3.9 Hz, 1H), 5.70 (d, *J = *3.9 Hz, 1H), 6.06 (s, 2H), 6.17 ( d, *J = *8.2 Hz, 1H), 6.98 (d, *J = *8.2 Hz, 1H), 7.86 (s, 1H).

*(3S)-3-((5R)-9-bromo-6-((1-(2-ethoxy-2-hydroxyethyl)-1H-1,2,3-triazol-4-yl)methyl)-4-methoxy-5,6,7,8-tetrahydro-[1,3]dioxolo[4,5-g]isoquinolin-5-yl)-6,7-dimethoxyisobenzofuran-1(3H)-one (5r, C*_28_ *H*_31_ *BrN*_4_ *O*_9_ *)*

 Yield: 70%, yellow powder, mixture of two isomers (52: 48), m.p.: 68-70^o^C, decompose, HRMS: [M+H]^+^ calcd= 675.1582, found= 677.2693, IR (KBr, cm^-1^): 3437, 2928, 1757, 1616, 1497, 1449, 728, ^1^H-NMR (300 MHz, CDCl_3_, mixture of two isomers (52: 48)) δ (ppm): 0.85-0.98 (m, 6H, mixture of two isomers), 1.36-1.44 (m, 4H, mixture of two isomers), 1.95-2.04 (m, 2H, mixture of two isomers), 2.27-2.34 (m, 2H, mixture of two isomers), 2.46-2.54 (m, 4H, mixture of two isomers), 3.44-2.51 (m, 4H, mixture of two isomers), 3.81 (d, *J = *13.8 Hz, 1H, minor), 3.78 (d, *J = *14.0 Hz, 1H, minor), 3.87 (s, 6H, mixture of two isomers), 3.99 (d, *J = *13.8 Hz, 1H, major), 3.90-3.97 (m, 6H, mixture of two isomers), 4.02 (d, *J = *14.0 Hz, 1H, major), 4.02-4.11 (m, 6H, mixture of two isomers), 4.47-4.55 (m, 6H, mixture of two isomers), 5.68 (d, *J = *3.6 Hz, 2H, mixture of two isomers), 6.06 (s, 4H, mixture of two isomers), 6.19 (d, *J = *8.1 Hz, 2H, mixture of two isomers), 6.99 (d, *J = *8.1 Hz, 2H, mixture of two isomers), 7.81 (s, 1H, minor), 7.86 (s, 1H, major).

*(3S)-3-((5R)-6-((1-(2-(allyloxy)-2-hydroxyethyl)-1H-1,2,3-triazol-4-yl)methyl)-9-bromo-4-methoxy-5,6,7,8-tetrahydro-[1,3]dioxolo[4,5-g]isoquinolin-5-yl)-6,7-dimethoxyisobenzofuran-1(3H)-one (5s, C*_29_ *H*_31_ *BrN*_4_ *O*_9_ *)*

 Yield: 70%, yellow powder, m.p.: 80-82 ^o^C, decomposed, HRMS: [M+H]^+^ calcd= 659.1269, found= 659.1365, IR (KBr, cm^-1^): 3434, 2923, 2855, 1755, 1612, 1499, 1450, 724, ^1^H-NMR (300 MHz, CDCl_3_, mixture of two isomers (52: 48)) δ (ppm): 1.98-2.04 (m, 4H, mixture of two isomers), 2.27-2.32 (m, 2H, mixture of two isomers), 2.49-2.54 (m, 4H, mixture of two isomers), 3.48-2.56 (m, 4H, mixture of two isomers), 3.78 (d, *J = *13.8 Hz, 1H, minor)**, **3.80 (d, *J = *13.8 Hz, 1H, major), 3.87 (s, 6H, mixture of two isomers), 3.99 (d, *J = *13.8 Hz, 1H, minor), 4.01 (d, *J = *13.8 Hz, 1H, major), 4.04 (s, 6H, minor), 4.07 (s, 3H, major), 4.08 (s, 3H, minor), 4.51 (m, 8H, mixture of two isomers), 5.16-5.24 (m, 2H, mixture of two isomers), 5.27-5.33 (m, 2H, mixture of two isomers), 5.65-5.69 (m, 2H, mixture of two isomers), 5.87-5.94 (m, 2H, mixture of two isomers), 6.06 (s, 4H, mixture of two isomers), 6.18 (d, *J = *8.0 Hz, 2H, mixture of two isomers), 6.99 (d, *J = *8.0 Hz, 2H, mixture of two isomers), 7.81 (s, 1H, major), 7.86 (s, 1H, minor). 

*(3S)-3-((5R)-9-bromo-6-((1-(2-hydroxybutyl)-1H-1,2,3-triazol-4-yl)methyl)-4-methoxy-5,6,7,8-tetrahydro-[1,3]dioxolo[4,5-g]isoquinolin-5-yl)-6,7-dimethoxyisobenzofuran-1(3H)-one (5t, C*_28_ *H*_31_ *BrN*_4_ *O*_8_ *)*

Yield: 60%, yellow powder, m.p.: 66-68 ^o^C, decomposed, HRMS: [M+H]^+^ calcd= 631.1320, found= 631.1406, IR (KBr, cm^-1^): 3442, 2926, 2856, 1754, 1621, 1499, 1448, 719, ^1^H-NMR (300 MHz, CDCl_3_, mixture of two isomers (50:50)) δ (ppm): 1.54-1.62 (m, 4H, mixture of two isomers), 1.070 (t, *J = *7.2 Hz, 6H, mixture of two isomers), 1.98-2.05 (m, 2H, mixture of two isomers), 2.19-2.25 (m, 2H, mixture of two isomers), 2.50-2.58 (m, 4H, mixture of two isomers), 3.53 (bp, 2H, mixture of two isomers), 3.73 (d, *J = *13.8 Hz, 1H, one isomer), 3.78 (d, *J = *14.0 Hz, 1H, one isomer), 3.87 (s, 6H, mixture of two isomers), 3.97 (d, *J = *13.8 Hz, 1H, one isomer), 4.01 (d, *J = *14.0 Hz, 1H, one isomer), 4.06 (s, 3H, one isomer), 4.07 (s, 6H, mixture of two isomers), 4.08 (s, 3H, one isomer), 4.38-4.45 (m, 6H, mixture of two isomers), 5.65 (s, 2H, mixture of two isomers), 5.70 (s, 2H, mixture of two isomers), 6.06 (s, 4H, mixture of two isomers), 6.15 (d, *J = *8.4 Hz, 2H, mixture of two isomers), 6.98 (d, *J = *8.4 Hz, 2H, mixture of two isomers), 7.83 (s, 1H, one isomer), 7.89 (s, 1H, one isomer).

*Biology*

*MTT assay on MCF-7 cell line*

The human MCF-7 cell line was supplied by the National Cell Bank of Iran (NCBI), Pasteur Institute of Iran (Tehran, Iran). It was prepared with 100 U/mL penicillin, 10 % fetal bovine serum, and 100 μg/mL streptomycin for forming DMEM (Dulbecco’s Modified Eagle Medium). This cell line was stabilized at 37 °C in atmosphere humidity with 5% CO_2_. Then, DMSO was used to dissolve the semi-synthetic derivatives of 9-bromonoscapine and making 1 mM stock. End concentrations were between 10–1000 µM with serum-free culture medium created by dilution.

*Method*

Cytotoxic effects were studied by the MTT assay. The 96-well plates with cell lines were prepared and put at 37 °C with 5% CO_2_ overnight in incubator. Suitable concentrations of 9-bromonoscapine analogs were added and kept for 24 hours. Finally, a serum of MTT at the end of the concentration (0.5 mg/mL) without medium was added to cell lines, and they were put in an incubator again for 4 hours. To measure IC_50_, the absorbance of the formazan crystals dissolved in the observed DMSO at 540-570 nm.

*Molecular Docking study*

All docking calculations were done in the ″Extra Precision″ (XP) mode of Glide docking by Schrodinger software. A 3.58 Å crystal structure of tubulin was downloaded from the PDB (PDB code:1SA0) (31). The grid box has been designed in 20×20×20 Å based sized of largest ligand in the active site of Colchicine. Ten more stable conformers of new synthetic compounds were selected to study their interaction in mentioned bonding site of tubulin.

## Results and Discussion

*Chemistry*

**Scheme 1:**
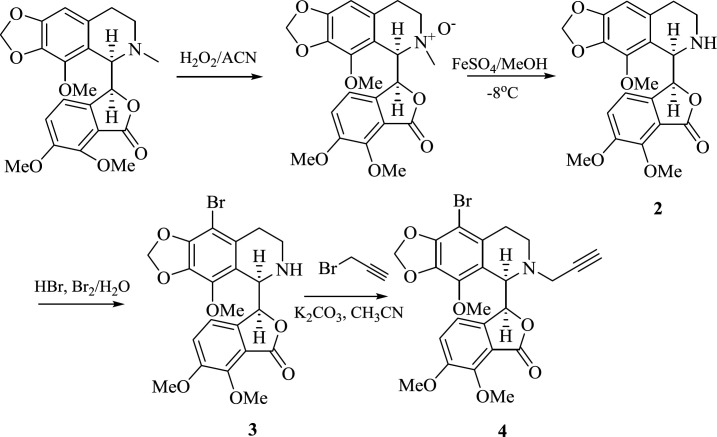
Synthesis of compounds 2-4

Our strategy for the synthesis of target molecules is depicted in Scheme 1. Nor-noscapine (**2**) was synthesized according to the literature procedure in the presence of hydrogen peroxide and ferrous sulfate (29, 30). Bromination of **2** by HBr/Br_2 _followed by propargylation of the secondary amine (**3**), furnished the terminal alkyne moiety as one of the key building blocks needed for the Huisgen’s 1,3-dipolar cycloaddition. 

**Scheme 2:**
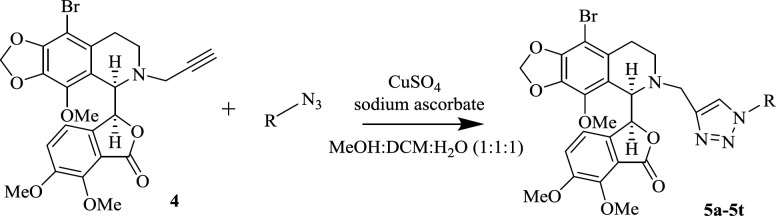
Synthesis of new 1,2,3-triazole-tethered noscapine derivatives

The next step for the synthesis of the target triazole-tethered noscapine derivatives, was the reaction of compound **4** with different azides to add the 1,2,3-triazole ring on the noscapine scaffold (Scheme 2). However, first we had to optimize the reaction conditions. Therefore, reaction solvent, catalyst percentage, and temperature were optimized. The results are summarized in Table 1. As can be seen, MeOH, DCM, and water with different ratios were used. Also, catalyst ranges between 10-15 mol% and temperatures between r.t. to 35 ^o^C were studied. Eventually, MeOH: DCM: H_2_O (1:1:1) and 15 mol% of the catalyst at room temperature were found to be the best conditions. 

With the optimized conditions in hand, the target molecules were synthesized in high yields. To investigate the effect of different substituents of triazole moiety on the biological impact of the products, a wide range of azides, including aromatic, benzylic, and aliphatic azides, were used (Table 2). The results are summarized in Table 3.

As can be seen, most of the compounds were synthesized in good to excellent yields. The first category was the 1,2,3-triazole derivatives with substituted aryl and heteroaryl rings at 1-position (**5a**-**5k**). The substituents included halogen (F, Cl, Br) and alkyl, methoxy, and nitro groups. 

The second class was those from the reaction of benzylic azides with the terminal alkyne group of **4** (**5l**- **5o**). The third group of the synthesized compounds. 

Those which aliphatic azidoalcohols were utilized as the starting material (**5p**- **5t**). These azides were synthesized from the nucleophilic ring-opening reaction of NaN_3_ with the corresponding epoxides (24).

*Cytotoxicity on MCF-7 cell line*

As can be seen in Table 3, most of the synthesized products revealed better cytotoxicity against MCF-7 cell line than nor-noscapine and 9-bromo-nor-noscapine as the parent compounds. Among different substituents on the triazole moiety, aromatic rings bearing chloro, methyl, methoxy, and nitro groups (**5b**, **5c**, **5g,** and **5h**) showed weaker activity than those of fluoro and compounds without any substituent (**5i**, **5j,** and **5d**). Also, benzylic model compounds showed promising activities in which unsubstituted ring (**5l**) was the best. Among the synthesized compounds, those with hydroxylated aliphatic side chains showed the highest cytotoxicities on the breast cancer cell line (**5p**,** 5q, **and** 5r**). It seems that size, orientation and the polar OH group capable of making hydrogen bonds are among the key factors of the observed activity of this class of compounds.

*The docking results*

To investigate the relation between structures of the synthesized compounds and their activities, a molecular docking study was made for fitting the target molecules into the active site of tubulin. The results are summarized in Table 4, which shows that there is a good correlation between the calculated activities with those of the *in-vitro* tests. 

**Figure 2:**
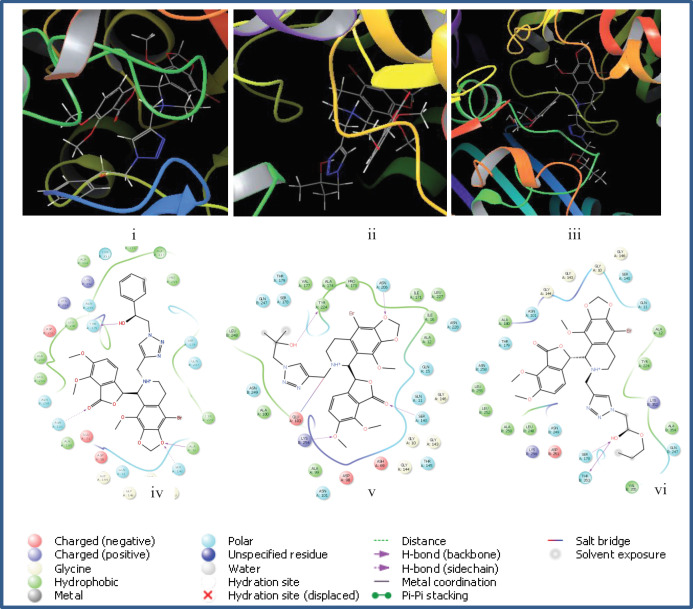
2D and 3D pictures of binding sites for compounds, 5p (i, iv), 5q (ii, v) and 5r (iii, vi) as the most effective ligands in tubulin active site.

It was observed that compounds **5p**, **5q,** and **5r **exhibited the highest docking scores (-8.047, -7.425 and -7.820 kcal/mol, respectively) which included an aliphatic side chain with a hydroxyl group. These compounds showed the best cytotoxicities against MCF-7 cell line. The first significant interaction was hydrogen bonding between the hydroxyl group with some polar amino acids residues. For **5p,** the OH group showed strong interactions with amino acids Thr179 and Asn249, while in **5q **and** 5r**, the hydrogen bonding occurred with Ser178, Thr353, and Tyr224 of the active site. These results showed the crucial role of the hydroxyl group in the cytotoxic effects of active compounds. Also, other oxygen atoms in the noscapine backbone of active compounds **5p, 5q, **and** 5r**, had hydrogen bond interaction with Lys254, Ser140, Asn101, and Asn258 (Figure 2). Compound **5p** had some hydrophobic interactions with Ala316, Ala317, and Met259. In addition, compounds **5f** and **5i** were noticeable ligands with docking scores equal to -7.154 and -7.173 Kcal/mol, respectively. In all new derivatives, the linker group was 1,2,3-triazole, showing some polar and hydrophilic interaction with Ser178 and Gln240 for **5p **and with Asn249 for **5q** and **5r**.

The 1:1 ratio of the synthesized derivatives with tubulin was consistent with previous reports, which showed the same stoichiometry (32). Noscapine itself has a hydrogen atom at 9 position which occupied an electron cloud with a size equal to 1.2 Å in Gaussian calculations. The previous docking studies showed that there is an empty space around the position 9 in the binding pocket of tubulin (33). If the hydrogen is substituted with halogen atoms such as bromine, the electron cloud size is increased to 1.81 Å and consequently fit better in the vicinity of this loop. To benchmark this hypothesis docking of the 9 analogs of **5p**, **5q** and **5r** was investigated and we observed that lower scores were obtained (-7.520, -6.771 and -6.228 kcal/mol, respectively) which confirmed this speculation. 

**Table 1:** Optimization of the click reaction condition.

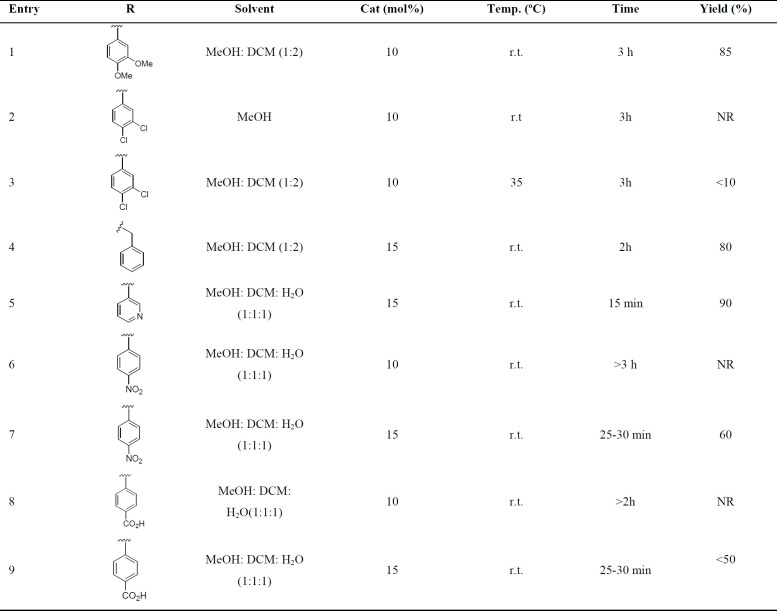

**Table 2:** Structures of applied azides

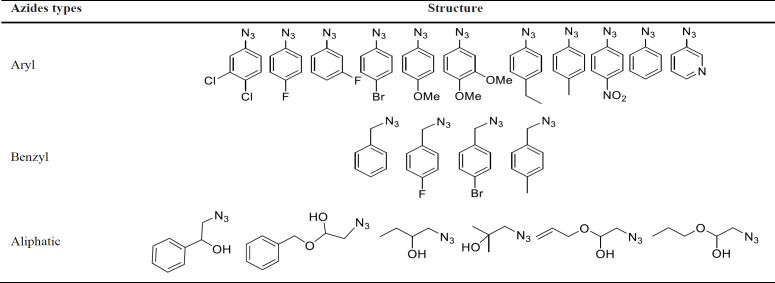

**Table 3:** Structure of the synthesized compounds and their IC_50_ values against MCF-7 cell line

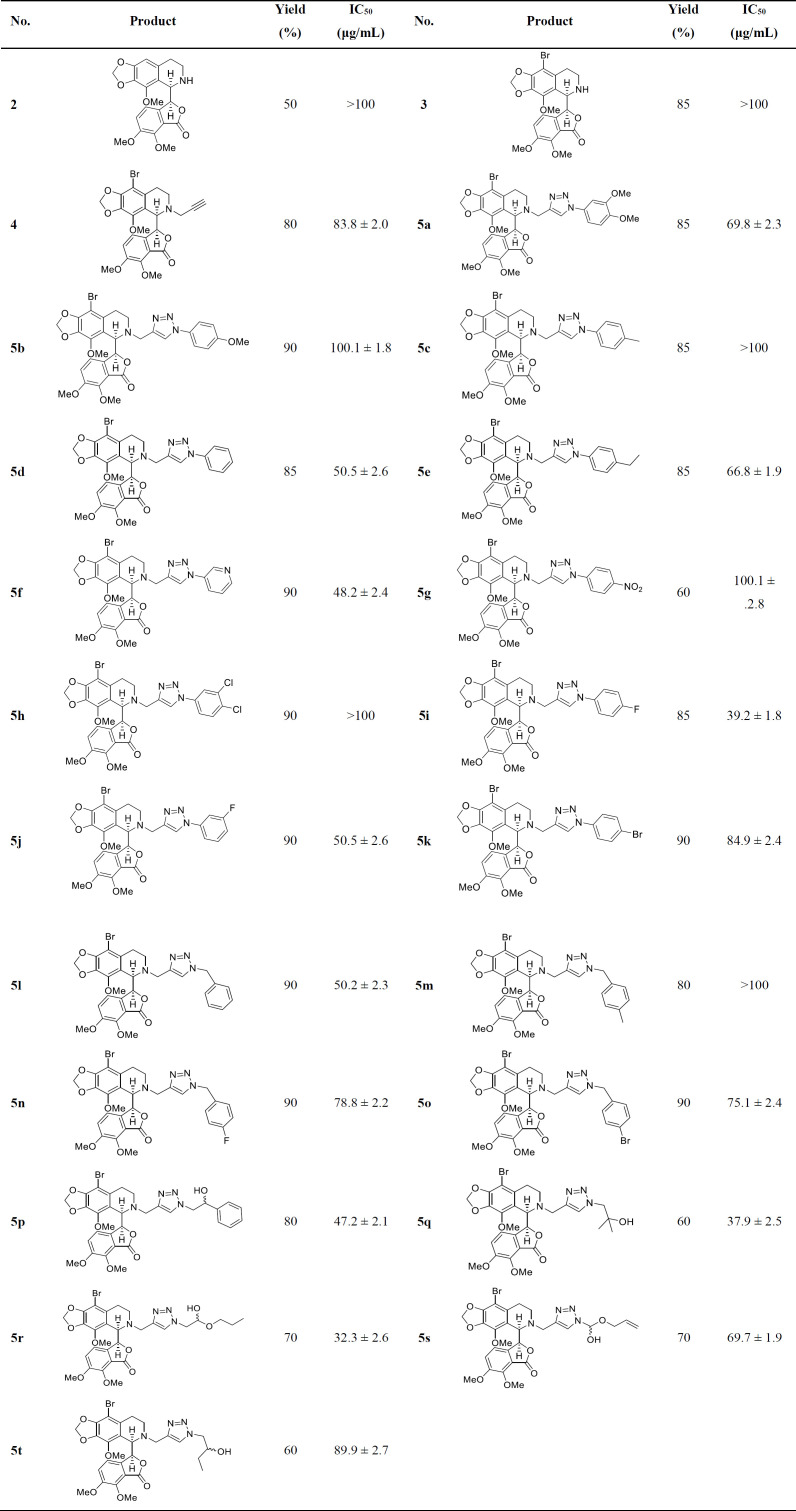

**Table 4:** Docking score data of the new noscapine derivatives with tubulin

**Com.**	**Docking score**	**Com.**	**Docking score**	**Com.**	**Docking score**
**1**	-6.850	**2**	-6.341	**3**	-6.079
**4**	-6.490	**5a**	-6.959	**5b**	-5.973
**5c**	-6.579	**5d**	-6.550	**5e**	-6.782
**5f**	-7.157	**5g**	-6.014	**5h**	-6.752
**5i**	-7.173	**5j**	-6.362	**5k**	-6.440
**5l**	-6.036	**5m**	**-7.737**	**5n**	-6.672
**5o**	-6.923	**5p**	-8.047	**5q**	-7.425
**5r**	-7.820	**5s**	-6.465	**5t**	-6.046
**Colchicine **	-7.888				

## Conclusion

In this paper, twenty-three novel derivatives of noscapine have been synthesized. Due to the reports about the high activity of 9-bromonoscapine compared to the parent molecule, 9-bromo-nor-noscapine has been selected as the lead compound in this project. Tethering substituted triazole rings with 9-bromonoscapine ended up with an increase in cytotoxicity of the target molecules. Among the synthesized compounds, those with an aliphatic side chain bearing an OH group showed the best cytotoxicities and the highest docking scores. Moreover, it was shown that the substitution of bromine at 9 position, made an increment in fitting strength of the molecules into the tubulin active site.

## References

[1] Winzer T, Gazda V, He Z, Kaminski F, Kern M, Larson TR, Li Y, Meade F, Teodor R, Vaistij FE (2012). A Papaver somniferum 10-gene cluster for synthesis of the anticancer alkaloid noscapine. Science..

[2] Mishra KB, Mishra RC, Tiwari VK (2015). First noscapine glycoconjugates inspired by click chemistry. RSC Adv..

[3] Ghaly PE, El-Magd RMA, Churchill CD, Tuszynski JA, West F (2016). A new antiproliferative noscapine analogue: chemical synthesis and biological evaluation. Oncotarget..

[4] Dahlström B, Mellstrand T, Löfdahl C-G, Johansson M (1982). Pharmakokinetic properties of noscapine. Eur. J. Clin. Pharmacol..

[5] Verma AK, Bansal S, Singh J, Tiwari RK, Sankar VK, Tandon V, Chandra R (2006). Synthesis and in vitro cytotoxicity of haloderivatives of noscapine. Bioorg. Med. Chem..

[6] Santoshi S, Naik PK, Joshi HC (2011). Rational design of novel anti-microtubule agent (9-azido-noscapine) from quantitative structure activity relationship (QSAR) evaluation of noscapinoids. J. Biomol. Screen..

[7] Zhou J, Panda D, Landen JW, Wilson L, Joshi HC (2002). Minor alteration of microtubule dynamics causes loss of tension across kinetochore pairs and activates the spindle checkpoint. J. Biol. Chem..

[8] Aneja R, Vangapandu SN, Lopus M, Viswesarappa VG, Dhiman N, Verma A, Chandra R, Panda D, Joshi HC (2006). Synthesis of microtubule-interfering halogenated noscapine analogs that perturb mitosis in cancer cells followed by cell death. Biochem. Pharmacol..

[9] DeBono A, Capuano B, Scammells PJ (2015). Progress toward the development of noscapine and derivatives as anticancer agents. J. Med. Chem..

[10] DeBono AJ, Xie JH, Ventura S, Pouton CW, Capuano B, Scammells PJ (2012). Synthesis and Biological Evaluation of N-Substituted Noscapine Analogues. ChemMedChem..

[11] Aggarwal S, Ghosh NN, Aneja R, Joshi H, Chandra R (2002). A Convenient Synthesis of Aryl-Substituted N-Carbamoyl/N-Thiocarbamoyl Narcotine and Related Compounds. Helv. Chim. Acta..

[12] Naik PK, Chatterji BP, Vangapandu SN, Aneja R, Chandra R, Kanteveri S, Joshi HC (2011). Rational design, synthesis and biological evaluations of amino-noscapine: a high affinity tubulin-binding noscapinoid. J. Comput. Aided Mol. Des..

[13] Porcù E, Sipos A, Basso G, Hamel E, Bai R, Stempfer V, Udvardy A, Bényei AC, Schmidhammer H, Antus S (2014). Novel 9′-substituted-noscapines: synthesis with Suzuki cross-coupling, structure elucidation and biological evaluation. Eur. J. Med. Chem..

[14] Nemati F, Bischoff-Kont I, Salehi P, Nejad-Ebrahimi S, Mohebbi M, Bararjanian M, Hadian N, Hassanpour Z, Jung Y, Schaerlaekens S (2021). Identification of novel anti-cancer agents by the synthesis and cellular screening of a noscapine-based library. Bioorg. Chem..

[15] Mishra RC, Karna P, Gundala SR, Pannu V, Stanton RA, Gupta KK, Robinson MH, Lopus M, Wilson L, Henary M (2011). Second generation benzofuranone ring substituted noscapine analogs: synthesis and biological evaluation. Biochem. Pharmacol..

[16] Zhou J, Gupta K, Aggarwal S, Aneja R, Chandra R, Panda D, Joshi HC (2003). Brominated derivatives of noscapine are potent microtubule-interfering agents that perturb mitosis and inhibit cell proliferation. Mol. Pharmacol..

[17] Nagireddy PKR, Kommalapati VK, Siva Krishna V, Sriram D, Tangutur AD, Kantevari S (2019). Imidazo [2, 1-b] thiazole-Coupled Natural Noscapine Derivatives as Anticancer Agents. ACS Omega.

[18] Harikandei KB, Salehi P, Ebrahimi SN, Bararjanian M, Kaiser M, Khavasi HR, Al-Harrasi A (2019). N-Substituted noscapine derivatives as new antiprotozoal agents: synthesis, antiparasitic activity and molecular docking study. Bioorg. Chem..

[19] Alijanvand SH, Christensen MH, Christiansen G, Harikandei KB, Salehi P, Schiøtt B, Moosavi-Movahedi AA, Otzen DE (2020). Novel noscapine derivatives stabilize the native state of insulin against fibrillation. Int. J. Biol. Macromol..

[20] M Heravi M, Tamimi M, Yahyavi H, Hosseinnejad T (2016). Huisgen’s cycloaddition reactions: A full perspective. Curr. Org. Chem..

[21] Kolb HC, Finn M, Sharpless KB (2001). Click chemistry: diverse chemical function from a few good reactions. Angew. Chem. Int. Ed..

[22] Tron GC, Pirali T, Billington RA, Canonico PL, Sorba G, Genazzani AA (2008). Click chemistry reactions in medicinal chemistry: Applications of the 1, 3-dipolar cycloaddition between azides and alkynes. Med. Res. Rev..

[23] Duan YC, Ma YC, Zhang E, Shi XJ, Wang MM, Ye X-W, Liu HM (2013). Design and synthesis of novel 1, 2, 3-triazole-dithiocarbamate hybrids as potential anticancer agents. Eur. J. Med. Chem..

[24] Salehi P, Babanezhad-Harikandei K, Bararjanian M, Al-Harrasi A, Esmaeili M-A, Aliahmadi A (2016). Synthesis of novel 1, 2, 3-triazole tethered 1, 3-disubstituted β-carboline derivatives and their cytotoxic and antibacterial activities. Med. Chem Res..

[25] Esmaeelzadeh M, Salehi P, Bararjanian M, Gharaghani S (2019). Synthesis of new triazole tethered derivatives of curcumin and their antibacterial and antifungal properties. J. Iran. Chem Soc..

[26] Khaligh P, Salehi P, Bararjanian M, Aliahmadi A, Khavasi HR, Nejad-Ebrahimi S (2016). Synthesis and in Vitro Antibacterial Evaluation of Novel 4-Substituted 1-Menthyl-1, 2, 3-triazoles. Chem. Pharm. Bull..

[27] Mohebbi M, Salehi P, Bararjanian M, Aliahmadi A, Safavi-Sohi R, Ghasemi JB (2014). Synthesis, antibacterial activity, and CoMFA study of new 1, 2, 3-triazolyl 7-carboxamidodesacetoxy cephalosporanic acid derivatives. Med. Chem. Res..

[28] Dabiri M, Salehi P, Bahramnejad M, Sherafat F (2010). Synthesis of diheterocyclic compounds based on triazolyl methoxy phenylquinazolines via a one-pot four-component-click reaction. J. Comb. Chem..

[29] Allen AC, Cooper DA, Moore JM, Gloger M, Neumann H (1984). Illicit heroin manufacturing by-products: capillary gas chromatographic determination and structural elucidation of narcotine-and norlaudanosine-related compounds. Anal. Chem..

[30] Nakano Y, Savage GP, Saubern S, Scammells PJ, Polyzos A (2013). A multi-step continuous flow process for the N-demethylation of alkaloids. Aust. J. Chem..

[31] Niu MM, Qin JY, Tian CP, Yan XF, Dong FG, Cheng ZQ, Fida G, Yang M, Chen H, Gu YQ (2014). Tubulin inhibitors: pharmacophore modeling, virtual screening and molecular docking. Acta Pharmacol. Sin..

[32] Ye K, Ke Y, Keshava N, Shanks J, Kapp JA, Tekmal RR, Petros J, Joshi HC (1998). Opium alkaloid noscapine is an antitumor agent that arrests metaphase and induces apoptosis in dividing cells. Proc. Natl. Acad. Sci. U. S. A..

[33] Aneja R, Lopus M, Zhou J, Vangapandu SN, Ghaleb A, Yao J, Nettles JH, Zhou B, Gupta M, Panda D (2006). Rational Design of the Microtubule-Targeting Anti–Breast Cancer Drug EM015. Cancer Res..

